# Crossover transseptal vasovasostomy: alternative for very selected cases of iatrogenic injury to vas deferens

**DOI:** 10.1590/S1677-5538.IBJU.2018.0445

**Published:** 2019-04-01

**Authors:** Fernando Korkes, Oseas Castro Neves

**Affiliations:** 1 Divisão de Urologia, Faculdade de Medicina do ABC, Santo André, SP, Brasil;; 2 Hospital Israelita Albert Einstein, São Paulo, SP, Brasil

**Keywords:** Azoospermia, Herniorrhaphy, Infertility, Vasovasostomy

## Abstract

Inguinal herniorraphy is a possible cause of iatrogenic seminal tract obstruction. Diagnosing and correcting these vasal injuries can be challenging. Successful re-anastomosis is technically challenging, with relatively low success rates. An uncommon alternative for selected cases is the crossover transseptal vasovasostomy. We herein report a case of a 36-year-old male patient with vas deferens injury after herniorraphy and a contralateral hypotrophic testis. He was successfully treated through microsurgical crossover transseptal vasovasostomy, with spontaneous pregnancy achieved, and the technique is presented in details.

## INTRODUCTION

Inguinal herniorraphy is a possible cause of iatrogenic seminal tract obstruction. Vas deferens injury, estimated to occur in 0.3% to 2.0% of herniorrhaphies, is not usually recognized at the time of hernia repair (1). If the patient sustains only a unilateral obstruction with a normal contralateral testicle and patent duct, the injury may remain unnoticed. However, if there is bilateral injury, a congenital absence of the contralateral vas or an abnormal contralateral testis, the patient will be rendered infertile (1–3).

Diagnosing and correcting these vasal injuries can be challenging. The obstruction is commonly associated with significant scarring at the inguinal region. Successful re-anastomosis is technically challenging, with relatively low success rates (4–7). An uncommon alternative for selected cases is crossover transseptal vasovasostomy (8–10). We herein report a case with successful application of this technique.

### Case report

A 36-year-old male patient with secondary infertility was admitted to our clinic. He had a 30-year-old wife with regular menstrual cycle and without gynecologic pathology on examination. He underwent right inguinal hernia repair three years prior to this first evaluation, after their first son was born. Sperm parameters were as follows: semen volume, 2.3cc; sperm count 3.0 × 10^6^/ mL; motility, 0%; and Kruger morphology 0%. Follicle stimulating hormone (FSH = 3.0mUi / mL), luteinizing hormone (LH = 3.9mUI / mL) and testosterone levels (350.1ng / dL) were within the normal range.

On his physical examination, right testicular volume was normal (11.7cc) and left testis was hypotrophic and retractile (7.3cc). No varicoceles could be detected neither on physical nor on ultrasound examination. His medical history did not reveal significant factors. Even though the patient wasn't azoospermic, the severe spermatic oligoasthenoteratospermia was incompatible with the prior pregnancy history. As the couple had a prior son and the only relevant factor was the inguinal hernia repair, it was suspected that a right vas deferens obstruction had occurred, and the associated left testicular atrophy was responsible for the low semen parameters. Facing the diagnosis, they decided to undergo intracytoplasmic sperm injection (ICSI); but after five unsuccessful procedures, with two unexplained miscarriages, the couple returned to attempt a surgical treatment.

**Figure 1:**
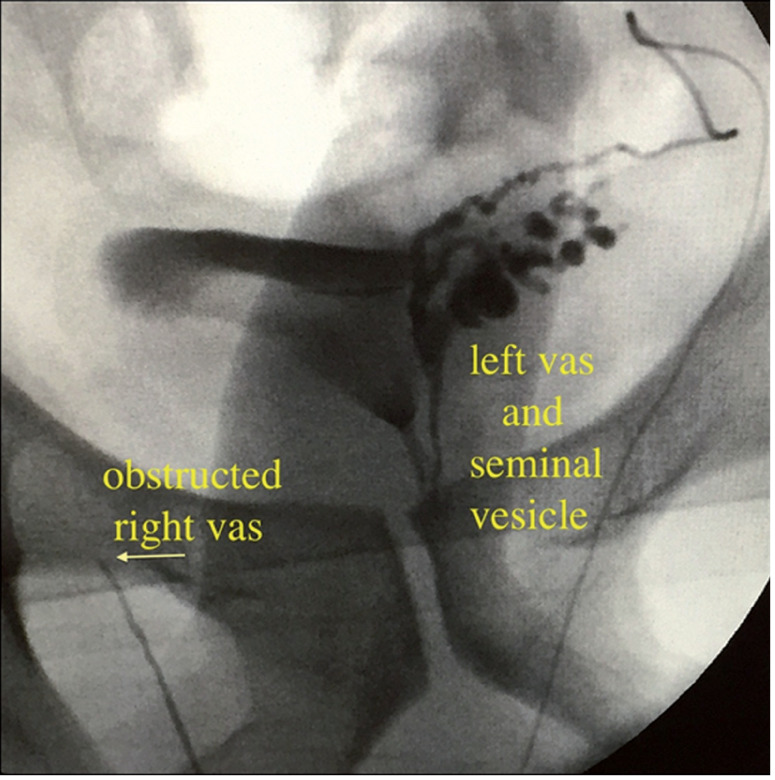
Intraoperative deferentography demonstrating obstructed right vas deferens and normal left vas and seminal vesicle.

**Figure 2:**
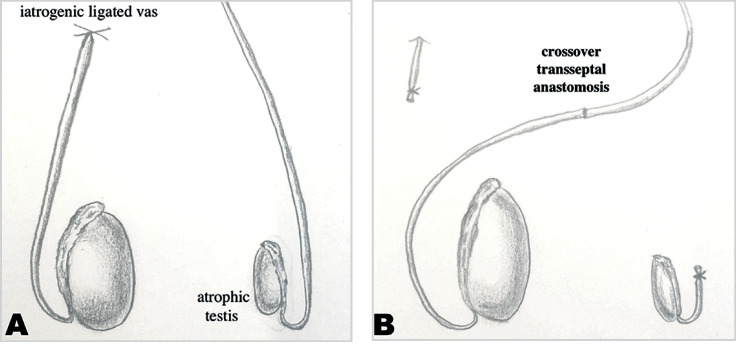
A) Schematic view of iatrogenic injury to right vas deferens with hypotrophic left testis. B) Schematic view of surgical procedure for microsurgical crossover transseptal vasovasostomy.

**Figure 3:**
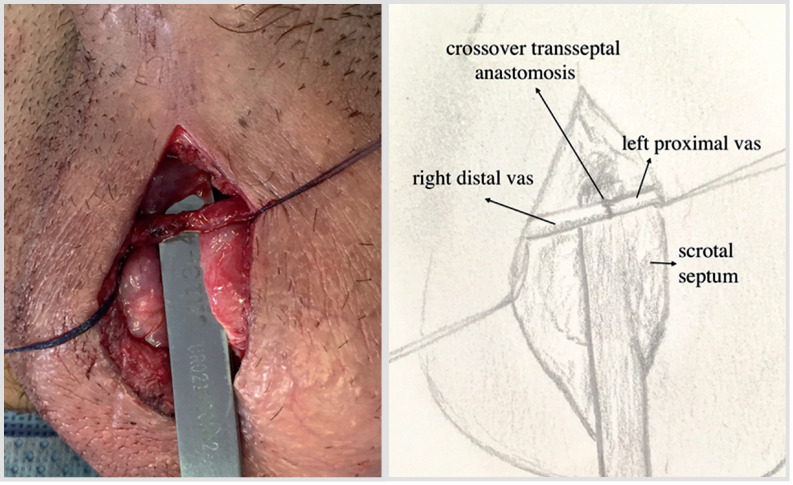
Final aspect of the microsurgical crossover transseptal vasovasostomy.

Surgical exploration of the testes was performed through a scrotal incision. After dissection of the vas deferens, a 23-gauge angiocatheter was placed into the lumen and contrast was injected to assure patency and confirm the diagnosis of obstruction (Figure-1). The catheter was placed in the segment planned to perform the anastomosis, assuring a long distal segment with the adequate (right) testicle and a long proximal segment in the side of the adequate (left) vas deferens (Figure-2A). After confirming the presence of spermatic fluid, a sufficient length of normal vas on the contralateral side was tunneled through an opening in the scrotal septum, and the anastomosis was performed. A 2-layer end-to-end microsurgical anastomosis with 9-0 suture was performed (Figures 2B and 3).

After one month of the procedure, a control sperm analysis was performed, and revealed: semen volume, 2.3cc; sperm count 17.0 × 10^6^/ mL; motility, 6%; and Kruger morphology 1%. After 3 months, the couple got pregnant and the baby was born healthy after an uneventful pregnancy.

## DISCUSSION

Inguinal hernia repair is one of the most common causes of iatrogenic vasal obstruction. The true incidence is unknown, but for a procedure performed over 20 million times annually worldwide, the risks become high (3, 7). The treatment of an inguinal hernia has changed considerably over the past decades. Polypropylene is the biomaterial most commonly used in hernia repair, and induces chronic inflammatory reaction, essential for optimal fixation and incorporation of the prosthesis. However, especially in the laparoscopic procedures that are mainly performed nowadays, the mesh is placed in close contact with the vas deferens, what can theoretically lead to an obstruction of these structures. Previous authors have reported several patients with postoperative obstructive azoospermia after hernia repair with polypropylene meshes (3–6, 8). This complication however can only be noted in men who undergo bilateral mesh repair for inguinal hernias and those who undergo a unilateral repair with impairment of contralateral testis. An additional factor is that fertility concerns might be present, what do not occur frequently in the elderly population that most commonly undergo such procedures. To better understand these risks, there is a prospective randomized trial currently being conducted (7).

When this unfortunate condition is suspected, there are limited diagnostic tools. If the vas is obstructed due to previous hernia repair, the epididymis may be thickened or indurated. Deferentography is conclusive although invasive, and normally performed intraoperatively. A testis biopsy can also confirm spermatogenesis on the obstructed side. Attempt to perform a vasovasostomy in the herniorraphy scarred tissue is cumbersome, with relatively low success rates (2, 4, 5). Distinctive complex approaches, including robotic anastomosis have also been described (8), and might be an alternative in bilateral cases. In the present case, there was only one adequately functioning testis, and a viable contralateral vas deferens. Even though rarely described, the crossover transseptal vasovasostomy can be a relatively straightforward alternative for these cases, as it is very similar to vasectomy reversal. Results therefore are expected to be similar to these commonly performed procedures. Even in a series in which crossover transseptal vasoepididymostomy was required, Sabanegh & Thomas reported a patency rate as high as an 89% (9).

Advances in assisted reproductive techniques and ICSI have brought a new treatment modality for men with obstructive azoospermia. However, it is associated to additional risks and costs. Female factors, noteworthy female age, have to be meticulously considered before choosing the best approach for a specific scenario. However, if there is a solitary functioning testis with irreparable ductal obstruction, crossover transseptal vasovasostomy can be an alternative to restore patency, and might be offered as an option to these couples interesting in achieving pregnancy.
